# 3D Visualization of the Temporal and Spatial Spread of Tau Pathology Reveals Extensive Sites of Tau Accumulation Associated with Neuronal Loss and Recognition Memory Deficit in Aged Tau Transgenic Mice

**DOI:** 10.1371/journal.pone.0159463

**Published:** 2016-07-28

**Authors:** Hongjun Fu, S. Abid Hussaini, Susanne Wegmann, Caterina Profaci, Jacob D. Daniels, Mathieu Herman, Sheina Emrani, Helen Y. Figueroa, Bradley T. Hyman, Peter Davies, Karen E. Duff

**Affiliations:** 1 Taub Institute for Research on Alzheimer's Disease and the Aging Brain, Columbia University Medical Center, New York, New York, United States of America; 2 Department of Pathology and Cell Biology, Columbia University Medical Center, New York, New York, United States of America; 3 Department of Neurology, Massachusetts General Hospital, Harvard Medical School, Charlestown, Massachusetts, United States of America; 4 Litwin-Zucker Center for Research in Alzheimer's Disease, Feinstein Institute for Medical Research, North Shore/LIJ Health System, Manhasset, New York, United States of America; Centre Hospitalier de l'Université Laval, CANADA

## Abstract

3D volume imaging using iDISCO+ was applied to observe the spatial and temporal progression of tau pathology in deep structures of the brain of a mouse model that recapitulates the earliest stages of Alzheimer’s disease (AD). Tau pathology was compared at four timepoints, up to 34 months as it spread through the hippocampal formation and out into the neocortex along an anatomically connected route. Tau pathology was associated with significant gliosis. No evidence for uptake and accumulation of tau by glia was observed. Neuronal cells did appear to have internalized tau, including in extrahippocampal areas as a small proportion of cells that had accumulated human tau protein did not express detectible levels of human tau mRNA. At the oldest timepoint, mature tau pathology in the entorhinal cortex (EC) was associated with significant cell loss. As in human AD, mature tau pathology in the EC and the presence of tau pathology in the neocortex correlated with cognitive impairment. 3D volume imaging is an ideal technique to easily monitor the spread of pathology over time in models of disease progression.

## Introduction

A combination of brain clearing and immunolabeling has recently been used to visualize amyloid and tau lesions in 3D in blocks of postmortem tissue from late stage human AD brain [1–3]. Additionally, amyloid deposits have been observed in intact mouse brain. However, the 3D visualization of tau pathology and the rigorous examination of how pathology distribution changes as the disease progresses has not been reported. Extracellular amyloid-β (Aβ) plaques and intraneuronal neurofibrillary tangles (NFTs) composed of hyperphosphorylated tau are the two major pathological hallmarks of AD [4]. The accumulation of abnormal (argyrophilic) tau starts in the transentorhinal cortex in the earliest stages of AD and spreads through the limbic and association cortices via the trisynaptic circuit in a precise and defined manner [4–6]. To create a mouse model that recapitulates the temporal and spatial spread of pathological tau along anatomically connected networks, we [7, 8] and others [9] have generated a transgenic mouse model (line EC-Tau) that differentially expresses an aggregating form of human tau at high levels in the hippocampal formation.

Traditional immunohistochemistry routinely provides a 2D picture of pathology with a limited depth of field, while 3D volume imaging of transparent brain enables the visualization of deep structures of the brain, which is very useful for studying changes in synaptic architecture and neuronal circuitry in the whole brain during disease progression [3]. Several tissue-clearing protocols have recently been published [10–18], which greatly accelerate the 3D visualization of the deep structures of large tissues or organs. iDISCO+, an improved version of iDISCO, is a simple and rapid method to immunolabel large tissue samples such as a whole mouse brain for volume imaging [18, 19]. In this study, we used the iDISCO+ method to investigate the temporal and spatial distribution of tau pathology in EC-Tau mice.

Previous studies characterizing the pathology, degeneration or cognitive behavior of the EC-Tau line have been limited to mice at less than 24 months of age. Mice at this age are at a relatively early stage of disease (Braak stage I-II), with the pathology restricted to the hippocampal formation, little extrahippocampal pathology and no neocortical pathology. Significant neuronal loss has been shown in the EC-II and parasubiculum in mice at 24 months of age [8], but cognitive function has only been tested in the EC-Tau line in mice up to 16 months of age [9, 20]. At this age they were reported to be cognitively normal. In this study, we not only show in 3D the areas that tau propagates to outside of the hippocampus, but also we identify areas with overt pathology such as the amygdala that had not been previously identified. Additionally, we show the intimate association with gliosis, pathology and neuronal loss, and importantly, we show the first evidence of memory deficit in this line.

## Materials and Methods

### Ethics Statement

This study was carried out in strict accordance with the recommendations in the Guide for the Care and Use of Laboratory Animals of the National Institutes of Health. The protocol was approved by the Committee on the Ethics of Animal Care and Use of the Columbia University (Protocol# AC-AAAN9950).

### Animals

The neuropsin-tTA “activator” line was crossed with the tetracycline-inducible TauP301L “responder” line (line rTg4510) to generate a tau transgenic mouse line with restricted expression of human full-length tauP301L (the EC-Tau line) [7, 21]. F1 offspring were used as experimental animals. All animals are maintained in the animal facility at the Columbia University Medical Center on a 12 h light/dark cycle with free access to food and water.

### Whole mount imaging using immunolabeling-enabled 3D imaging of solvent-cleared organs (iDISCO+)

Mice were anesthetized with ketamine (Henry Schein Animal Health, Dublin, OH, USA) and xylazine (Akorn, Decatur, IL, USA) and transcardially perfused with phosphate-buffered saline (PBS) (Fisher Scientific, Fair Lawn, New Jersey, USA) and then 4% paraformaldehyde (PFA) (Electron Microscopy Sciences, Hatfield, PA, USA). Mouse brains were harvested and fixed in 4% PFA in PBS at 4°C overnight. Mouse brain hemispheres were dehydrated, bleached, rehydrated and blocked as described in the iDISCO+ method [18, 19] (http://idisco.info/idisco-protocol/). Mouse brain hemispheres (EC-Tau: 8 mo, 1 male; 14 mo, 2 males; 24 mo, 2 females; 34 mo, 1 male; Neuropsin-tTA: 33 mo, 1 female; Tg4510: 16 mo, 1 male) were immunolabeled with Alexa Fluor 647-labeled CP27 antibody against human tau for 4.5 days at 37°C, then they were cleared with dibenzyl ether as previously described [18, 19]. The transparent mouse brain hemispheres were imaged using light sheet microscopy (Ultramicroscope II, LaVision Biotec) equipped with a sCMOS camera (Andor Neo) and a 23/0.5 objective lens equipped with a 6 mm working distance dipping cap. Z-stack images taken from light sheet microscopy were subjected to 3D rendering via Imaris 8.0 software (Bitplane). 3D animations were used to generate the movie using iMovie for Mac.

### 2D Immunohistochemistry

Mouse brains were harvested and drop-fixed in 4% PFA at 4°C overnight, followed by incubation in 30% sucrose (Sigma-Aldrich, Saint Louis, MO, USA). OCT-embedded brains were sectioned (35 μm) throughout on a horizontal plane with a cryostat (Leica CM3050S, Leica Biosystems, Buffalo Grove, IL, USA), and collected in individual wells. Every sixth free-floating sections (n = 12 per EC-Tau mouse, 2 EC-Tau mice at each age, 1 male and 1 female) starting from Bregma -2.04 mm to Bregma -4.88 mm (Paxinos G, mouse brain atlas, 2001) were selected and stained with mouse anti-tau MC1 antibody (1:1000), which specifically detects an abnormal conformational epitope of human tau that is associated with neurofibrillary tangle (NFT) formation [22]. Similarly, every 9th free-floating sections (n = 10 per mouse, 4 male and 1 female nontransgenic controls, 5 male EC-Tau mice at ~34 months of age) were selected and stained with mouse anti-NeuN antibody (EMD Millipore, Billerica, MA, USA; 1:1000), which is a neuronal marker. In addition, representative free-floating sections (n = 3 per mouse) from the control and EC-Tau mice at different ages (n = 4 mice per group at each age, 2 males and 2 females) were stained with mouse MC1 (1:1000), rabbit anti-IBA-1 (a microglial marker) (Wako, Richmond, VA, USA; 1:500), rabbit anti-CD68 (an activated microglial marker) (Abcam, Cambridge, MA, USA; 1:500), and rabbit anti-GFAP (an astrocyte marker) (Sigma-Aldrich; 1:8000), respectively. Immunolabeling was performed as previously described [7]. To demonstrate the distribution of MC1-positive human tau protein, images of 12 intact sections from each mouse were captured under 4x objective and stitched together using Photoshop CS5 (Adobe Systems, San Jose, CA, USA). The missing places in each stitched image were filled out manually.

### Fluorescent in situ hybridization (FISH) and coimmunofluorescence (Immuno-FISH)

Mice at 30 months of age (EC-Tau: 1 male and 1 female; nontransgenic control: 1 female; Tg4510 without tTA: 1 male) were anesthetized with ketamine and xylazine and transcardially perfused with nuclease-free PBS. Mouse brains were harvested and drop-fixed in 4% PFA in nuclease-free PBS at 4°C overnight, followed by incubation in 30% sucrose in nuclease-free PBS for cryoprotection. The brains were embedded in OCT compound and sectioned horizontally (10 μm thick) with a cryostat. Frozen sections were mounted on pre-cleaned SuperfrostPlus microscope slides (Fisher Scientific) and stored at -80°C.

Riboprobe templates corresponding to the 3’ untranslated region of human *MAPT* (NM_016835; nucleotides 2773–3602) were generated by RT-PCR from human brain tissue. XbaI and XhoI sites were added (with three extra nucleotides to improve endonuclease efficiency) to the 5’ ends of the primers (5’-ACGTCTAGA CAGTGATGGGAGTAAGAG-3’ and 5’-AGACTCGAGCTGGTTAGCCCTAAAGTC-3’). The amplified sequence was subcloned into Bluescript vector (pBluescript SK-; Stratagene, La Jolla, CA, USA), and the PCR product was purified and used for digoxigenin (DIG)-labeled cRNA probe generation [8]. DIG-labeled cRNA probes were used for FISH as previously described [23]. FISH with mouse anti-human Tau13 (BioLegend, San Diego, CA, USA; 1:1000) co-immunofluorescence was performed on 10-μm-thick, PFA-fixed frozen sections as previously described [8, 24]. The DIG probe was detected using sheep anti-DIG-POD Fab fragments (Roche, Indianapolis, IN, USA), followed by amplification using Alexa Fluor488 tyramide (Life Technologies, Grand Island, NY, USA). The Tau13 antibody was detected using preadsorbed biotin-labeled goat anti-mouse IgG1 (Abcam), followed by incubation with Alexa Fluor 647 streptavidin (Life Technologies). The sections were incubated with 0.3% Sudan black (Sigma-Aldrich) in 70% ethanol for 6 min to quench autofluorescence of lipofuscin and rinsed quickly in 70% ethanol. Following three washes with 0.02% Tween-20 (Sigma-Aldrich) in PBS [25], the sections were mounted with SlowFade gold anti-fade reagent (Molecular Probes, Eugene, OR, USA). Immuno-FISH images were taken using confocal laser scanning microscopy via Z-stack (LSM700, Zeiss, Thornwood, NY, USA).

### Immunofluorescence

Free-floating brain sections were prepared in the same way as for immunohistochemistry. Sections were blocked in PBST containing 5% normal goat serum for 30 min at room temperature, and incubated with both mouse anti-tau CP27 (a human specific tau antibody) (1:1000) and rabbit anti-MAP2 (a specific neuronal marker) (Abcam; 1:1000), rabbit anti-IBA-1 (1:500) or rabbit anti-GFAP (1:5000) antibodies in PBST containing 5% normal goat serum at 4°C overnight. For IBA-1 labeling, 10 μg/ml rat anti-mouse FcR block (BD Biosciences) was added at the block stage. After three washes with PBST, sections were incubated with Alexa Fluor 488 goat anti-mouse and Alexa Fluor 594 goat anti-rabbit IgG (Life Technologies; 1:500) for 1 h at room temperature on a rotator. Following three washes with PBS, autofluorescence in sections were quenched with 0.3% Sudan black in 70% ethanol (Decon Laboratories, King of Prussia, PA, USA) for 6 min at room temperature. The sections were rinsed with 70% ethanol and washed three times with 0.02% Tween-20 in PBS [25]. The nuclei were stained with 5 μg/ml Hoechst33342 (Sigma-Aldrich) in PBST for 10 min at room temperature. Following three washes with PBS, sections were mounted on slides using SlowFade gold anti-fade reagent and imaged using confocal laser scanning microscopy via Z-stack.

### Neuronal counts

A semiquantitative count of NeuN^+^ neurons in the EC-II, EC-III/IV, pre-/para-subiculum (PPS), subiculum (Sub), CA1, and dentate gyrus (DG) was performed in the above selected sections from 5 nontransgenic controls and 5 EC-Tau mice. For each mouse, a total of 10 NeuN stained horizontal sections starting from Bregma -2.04 mm, spaced at 300 μm, were included for automated cell counting (http://imagej.net/Particle_Analysis) using the ImageJ software (version 1.48, US National Institutes of Health, Bethesda, Maryland, USA).

### Novel object recognition (NOR) test

The NOR task was performed by an experimenter blind to the treatments of the animals as previously described [26]. The day prior to training, mice (n = 5 male and 3 femalenontransgenic controls, and 6 male and 2 female EC-Tau mice at 30+ months of age) were habituated to experimental apparatus consisting of a white rectangular open field (60 cm x 50 cm x 26 cm) for 5 min in the absence of any objects. On the second day, mice were placed in the experimental apparatus in the presence of two identical rectangle objects and allowed to explore them for 10 min. After a retention interval of 24 h, mice were placed again in the apparatus, where one of the rectangle objects was replaced by a novel circle object. In both training and test tasks, objects and the apparatus were rinsed with ethanol between trials and before the first trial. Exploration of the objects was defined as the mice facing and sniffing the objects within 2-cm distance and/or touching them. All training and testing sessions were recorded using automated, ANY-maze video tracking software. The ability of the mouse to recognize the novel object was determined by dividing the mean time exploring the novel object by the mean of the total time exploring the novel and familiar objects during the test session. This value was multiplied by 100 to obtain a percentage preference for the novel object (T_novel_/[T_novel_ + T_familiar_] × 100).

### Statistical analysis

Prism 4 software (GraphPad, San Diego, CA, USA) was used to analyze the data. All the data are expressed as mean ± the standard error of the mean (SEM). Student’s *t*-test was used to analyze the neuronal counting. The non-parametric Mann-Whitney *U* test was used to compare the data of gliosis and novel object recognition test. A value of *p* < 0.05 was considered statistically significant.

## Results

### Visualization of human tau distribution in EC-Tau mice in 3D

**Fig 1:**
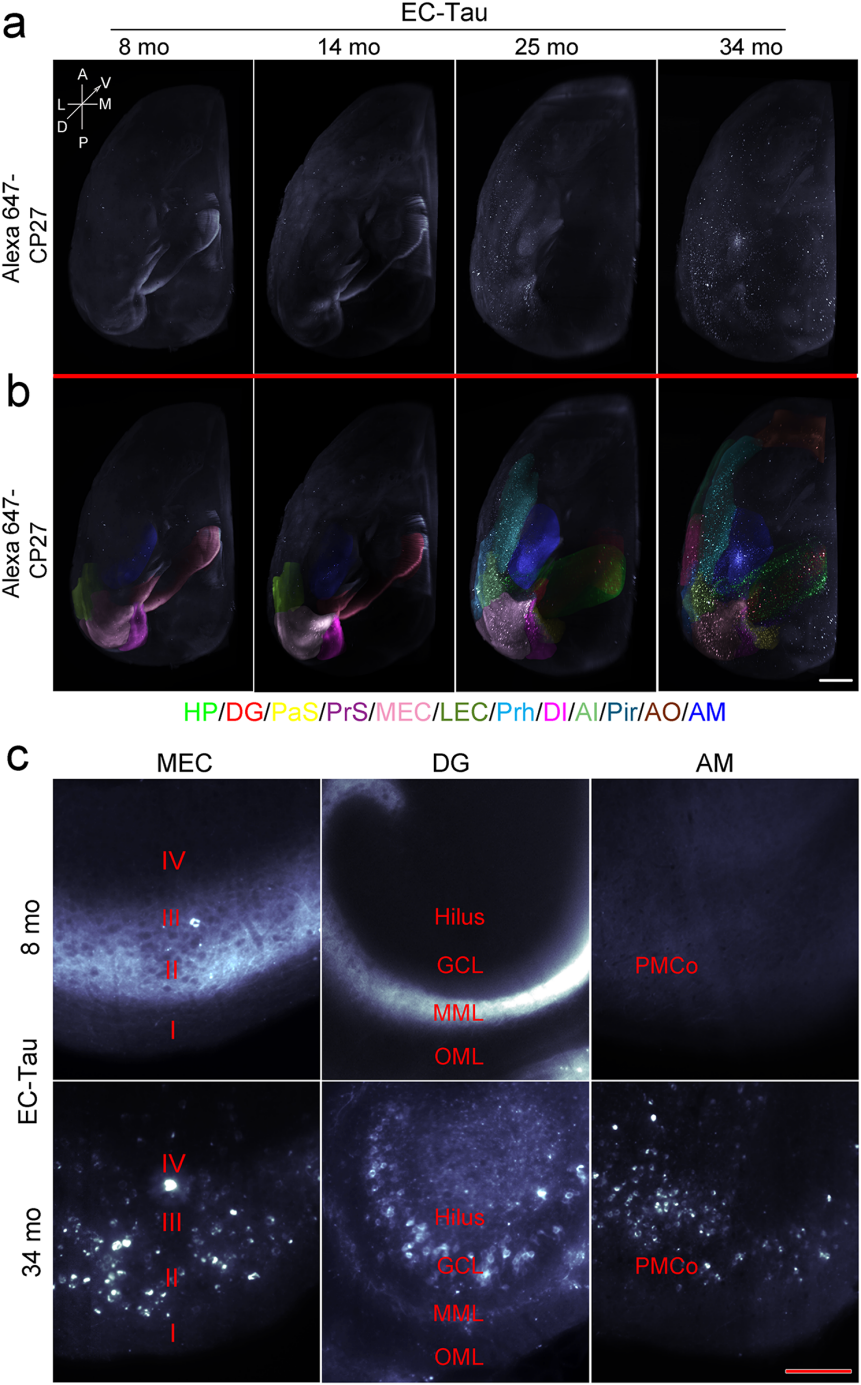
Visualization of tau pathology in EC-Tau mice in 3D. Hemi brains from 8, 14, 25 and 34-mo-old EC-Tau mice was stained with Alexa647-labeled CP27 antibody and cleared using iDISCO+ method. The transparent mouse brains were imaged using light sheet microscopy, and Z-stack images were subjected to 3D rendering process using Imaris. (a) Representative snapshots of 3D images of tau staining in EC-Tau mice. Tau immunoreactivity is shown in white. The orientation of the hemi brain was labeled as A, anterior; P, posterior; L, lateral; M, medial; D, dorsal; V, ventral. (b) Different brain regions with tau pathology are indicated by superimposed artificial colors. The regions with no or sparse tau immunoreactivity are not colored. HP, hippocampus; DG, dentate gyrus; PaS, parasubiculum; PrS, presubiculum; MEC, medial entorhinal cortex; LEC, lateral entorhinal cortex; Prh, perirhinal cortex; DI, dysgranular insular cortex; AI, agranular insular cortex; Pir, piriform cortex; AO, anterior olfactory area; and AM, amygdala. Scale bar = 1 mm. Movies are provided in supplemental material. (c) Representative horizontal views of tau immunoreactivity in the MEC, DG and AM. I-IV, MEC layers; OML, outer molecular layer; MML, middle molecular layer; GCL, granule cell layer; and hilus of the DG; PMCo, posteromedial cortical amygdaloid area of AM. Scale bar = 150 μm.

**Fig 2:**
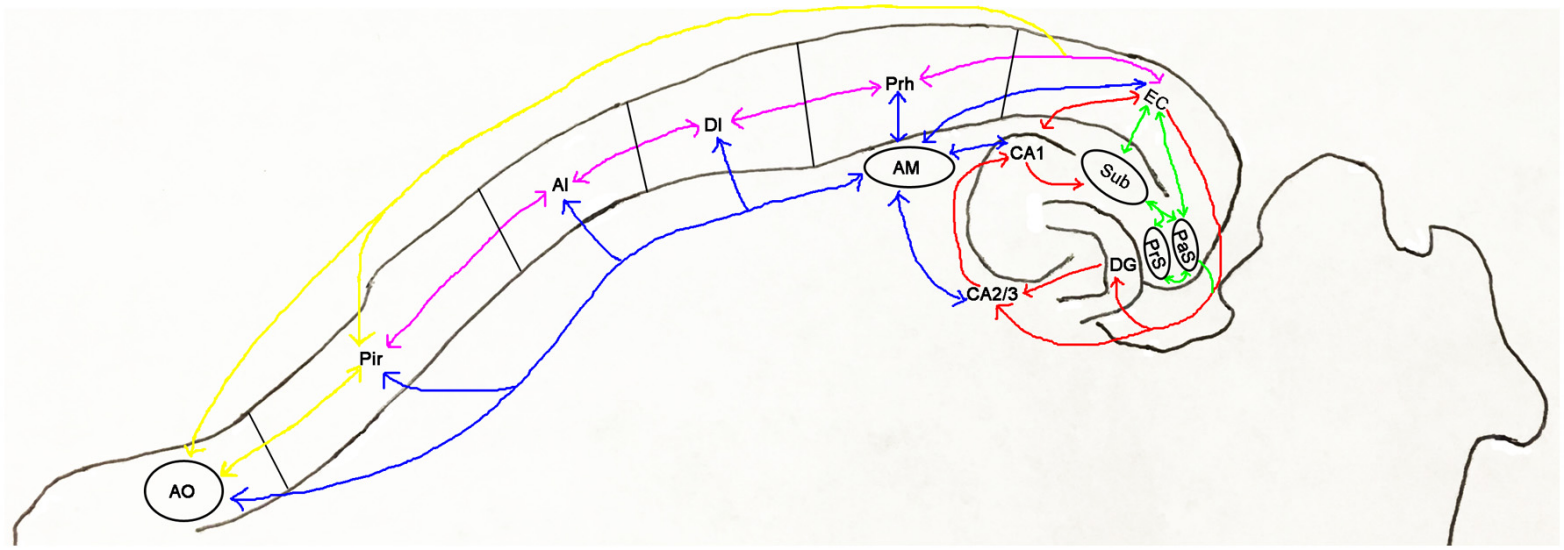
Schematic representation of the anatomical connections between entorhinal cortex and other brain regions in the mouse brain. The connections between EC and hippocampal regions (DG, CA1, CA2/3) are indicated by red arrows; EC and parahippocampal regions (Sub, subiculum; PaS; PrS) by green arrows; EC and neocortical regions (Prh, DI, AI) by magenta arrows; EC and olfactory system (Pir and AO) by yellow arrows; AM and other brain regions by blue arrows. The temporal and spatial distribution of tau pathology found in EC-Tau mice seems to follow the anatomical connections above.

At 8 months of age, 3D imaging of cleared EC-Tau mouse brain hemispheres revealed that most of the CP27 labeled human tau was in the form of nongranular axonal staining in the EC (layer II and III), parasubiculum (PaS), and the middle molecular layer (MML) of the dentate gyrus (DG). At this age, very few neurons showed somatodendritic granular human tau and they were restricted to the MEC and PaS (Fig 1 and S1 Movie for 3D representation). By 14 months of age, more neurons in the MEC and PaS had accumulated somatodendritic tau, and very few CP27 immunoreactive neurons were apparent in the granule cell layer (GCL) of the DG, or the pyramidal layer of the CA1 (Fig 1 and S2 Movie). By 25 months of age, human tau was mostly somatodendritic and it was observed in the EC, PaS, PrS, Sub, hippocampus (HP), the GCL of DG, anterior cingulate cortex (ACC), perirhinal cortex (Prh), and piriform cortex (Pir) (Fig 1 and S3 Movie). By 34 months of age, somatodendritic human tau was observed in neurons in the neocortical regions and olfactory system including dysgranular insular cortex (DI), agranular insular cortex (AI), piriform cortex (Pir), and anterior olfactory area (AO) (Fig 1 and S4 Movie). There was no detectable CP27 tau staining in control mice (the responder line Tg4510 without tTA) (S5 Movie) or the activator line Neuropsin-tTA without Tg4510) (S6 Movie). Interestingly, very strong somatodendritic human tau immunoreactivity was observed in the amygdala (AM), especially in the posteromedial cortical amygdaloid area of 34-mo-old EC-Tau mice in the 3D images (Fig 1). Granular tau aggregation in the amygdala was scarcely seen in the 3D images at 8 or 14 months of age, but some was observed by 25 months of age. The temporal and spatial distribution of tau pathology seems to follow the anatomical connections between EC and other brain regions including hippocampus (red arrows), parahippocampal areas (green arrows), neocortex (magenta arrows), olfactory system (yellow arrows), and amygdala (blue arrows) (Fig 2).

### Confirmation of human tau distribution in EC-Tau mice in 2D

**Fig 3:**
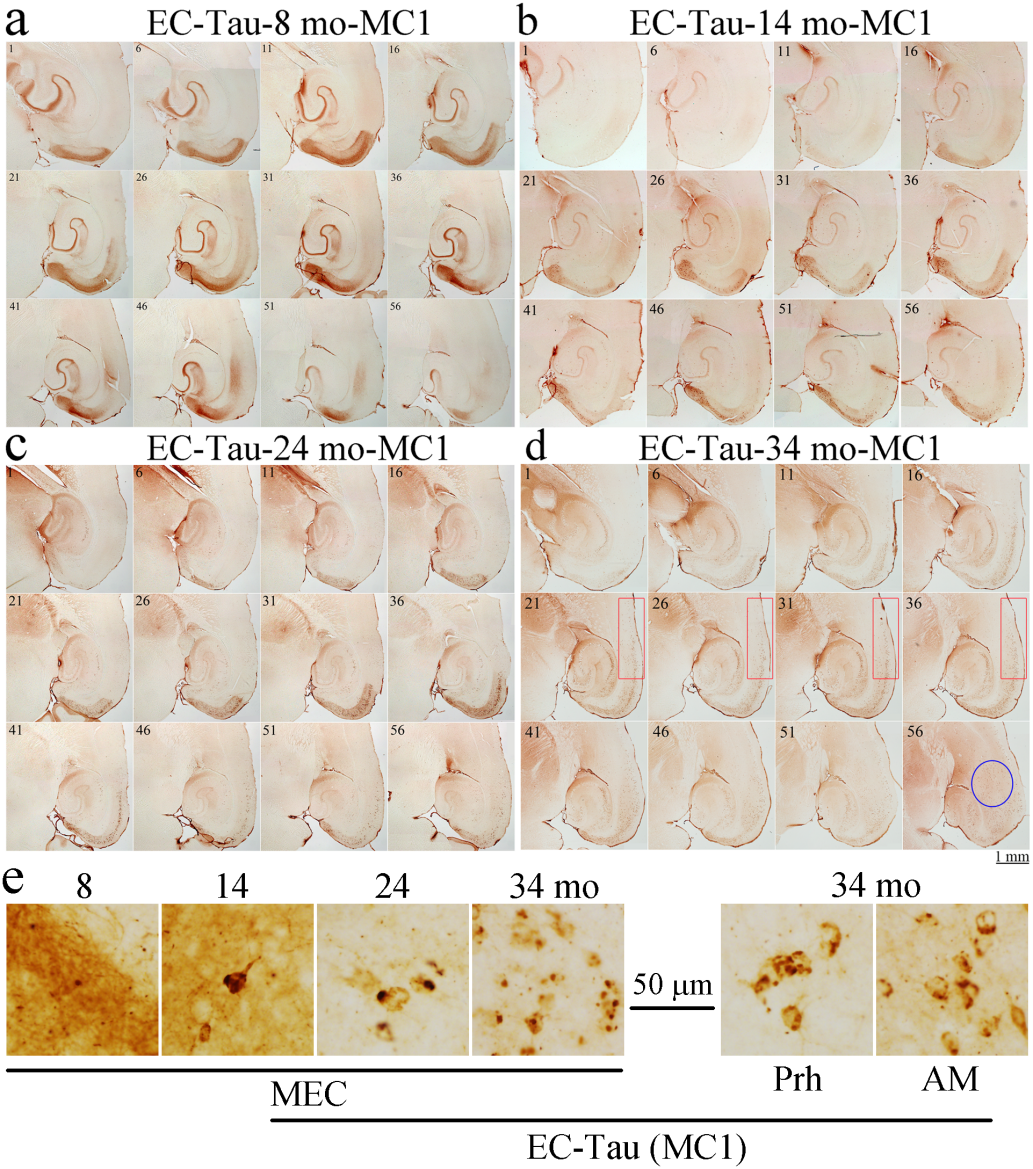
The spatial and temporal distribution of human tau in EC-Tau mice in 2D. Every sixth free-floating mouse brain section (35 μm) (sections 1, 6, 11, …, 56 per mouse, 2 mice at each age) starting from Bregma -2.04 mm to Bregma -4.88 mm was selected from 8-, 14-, 24- and 34-mo-old EC-Tau mice (a-d), stained with human specific pathological tau antibody (MC1) and developed using DAB as chromagen as described in the Materials and Methods. Tau immunoreactivity was indicated as brown staining. Images taken from each section were stitched together using Photoshop CS and presented as shown in the figure for comparison. The perirhinal cortex (Prh, highlighted in the red rectangles) and amygdala (AM, highlighted in the blue oval) of EC-tau mice had abundant MC1^+^ tau staining at 34-months of age, but not at younger ages. Scale bar = 1 mm. (e) Representative high magnification images of MC1 tau staining in MEC, Prh and AM. MEC, medial entorhinal cortex; Prh, perirhinal cortex; AM, amygdala. Scale bar = 50 μm.

**Fig 4:**
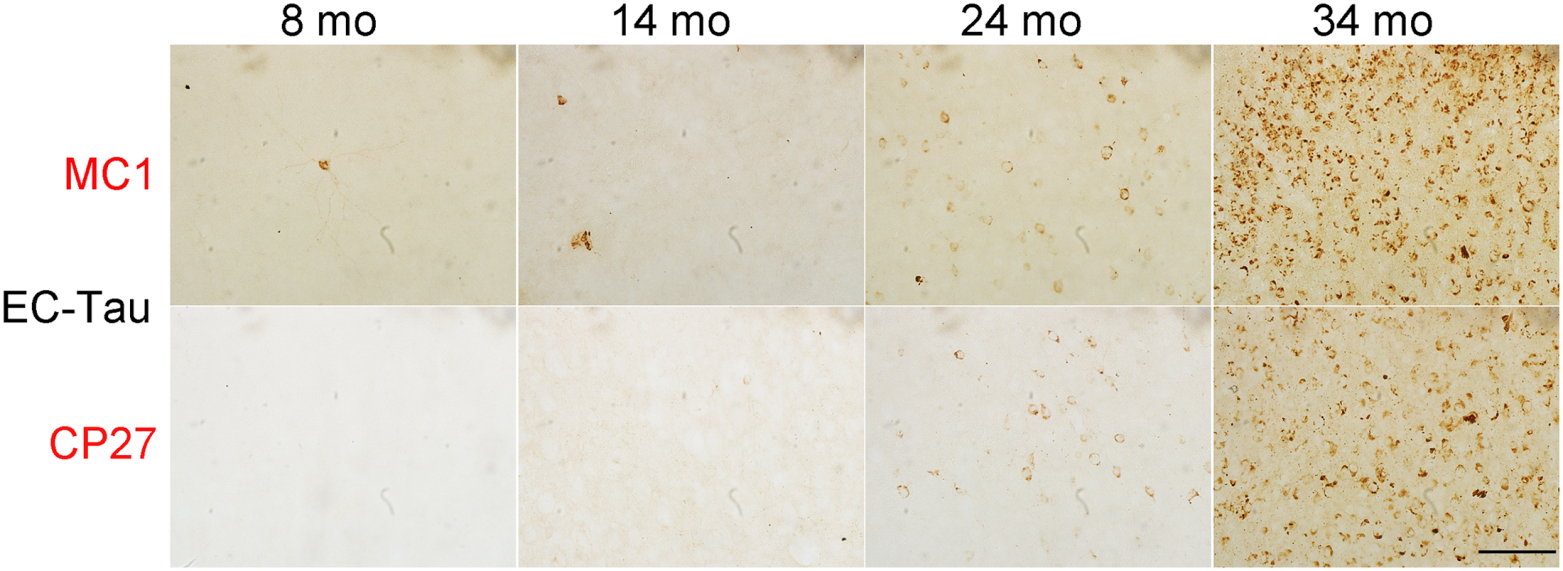
Tau pathology is observed in the amygdala in old EC-Tau mice. Mouse brain sections (n = 2 sections per animal, 3 animals per age) covering the amygdala area were stained with MC1 and CP27 anti-human tau antibodies as described in Materials and Methods to confirm the presence of human tau in this region. Scale bar = 100 μm.

To confirm the distribution of tau pathology revealed by 3D volume imaging we performed immunohistochemistry on 8, 14, 24, and 34-month-old EC-Tau mice (12 sections at each age) using the pathological human tau antibody MC1. Similar to 3D images with CP27 antibody, at 8 months of age, most MC1 immunoreactive (MC1^+^) tau was nongranular and restricted to neurites (axons) in superficial layers II and III of the EC, PaS, and axon terminals in the MML of DG, with very little tau staining in the somatodendritic compartments (Fig 3a and 3e) [7]. By 14 months of age, axonal MC1^+^ tau was reduced, but more tau was present in cell bodies in the EC-II and PaS. A few MC1^+^ cell bodies were observed in the GCL of the DG and the pyramidal layer of CA1 (Fig 3b and 3e) indicating that human tau had started to spread from the EC to the DG and CA1 via the performant path [7]. By 24 months of age, axonal MC1^+^ tau was dramatically reduced, and much more human tau protein had accumulated in cell bodies in the EC and hippocampal formation (DG, CA1, CA2/3, Sub and PrS) as well as in a few neurons in the perirhinal cortex (Prh) (Fig 3c and 3e). By 34 months of age, axonal MC1^+^ tau was almost undetectable and the protein had accumulated in cell bodies throughout the hippocampal formation and the parahippocampal regions including the Prh (red rectangles in Fig 3d and 3e) as well as other brain areas such as AO (S1 Fig) and AM (blue oval in Fig 3d and 3e). To confirm that tau pathology was present in the amygdala, MC1 and CP27 antibodies were used to label sections by traditional 2D immunohistochemistry. The results showed that the density and intensity of MC1 and CP27 somatodendritic staining in this region had increased dramatically in mice aged 24 and 34 months compared to younger EC-Tau mice at 8 to 14 months of age (Fig 4). MC1 or CP27 labeled tau was negligible in age and gender-matched nontransgenic control mice (data not shown). A qualitative assessment of the density and cellular distribution of human tau in the EC-tau line at different time points, and in different brain regions is shown in Table 1.

**Table 1:** Summary of the temporal and spatial distribution of tau pathology in EC-Tau mice.

Regions	8 mo	14 mo	24 mo	34 mo
EC axon	+++	++	+	-
PaS axon	+++	++	+	-
EC soma	-/+	+	++	+++
PaS soma	-/+	+	++	+++
DG soma	-	+	++	+++
CA soma	-	+	++	+++
Sub soma	-	+	++	+++
PrS soma	-	+	++	+++
Prh soma	-	-	+	++
DI soma	-	-	-/+	++
AI soma	-	-	-/+	++
Pir soma	-	-	+	++
AO soma	-	-	-/+	++
AM soma	-/+	-/+	+	+++

Note: EC = entorhinal cortex; PaS = parasubiculum; DG = dentate gyrus; CA = hippocampus; Sub = subiculum; PrS = presubiculum; Prh = perirhinal cortex; DI = dysgranular insular cortex; AI = agranular insular cortex; Pir = piriform cortex; AO = anterior olfactory area; AM = amygdala. -, none; -/+, very few; +, low, ++, medium; +++, high.

**Fig 5:**
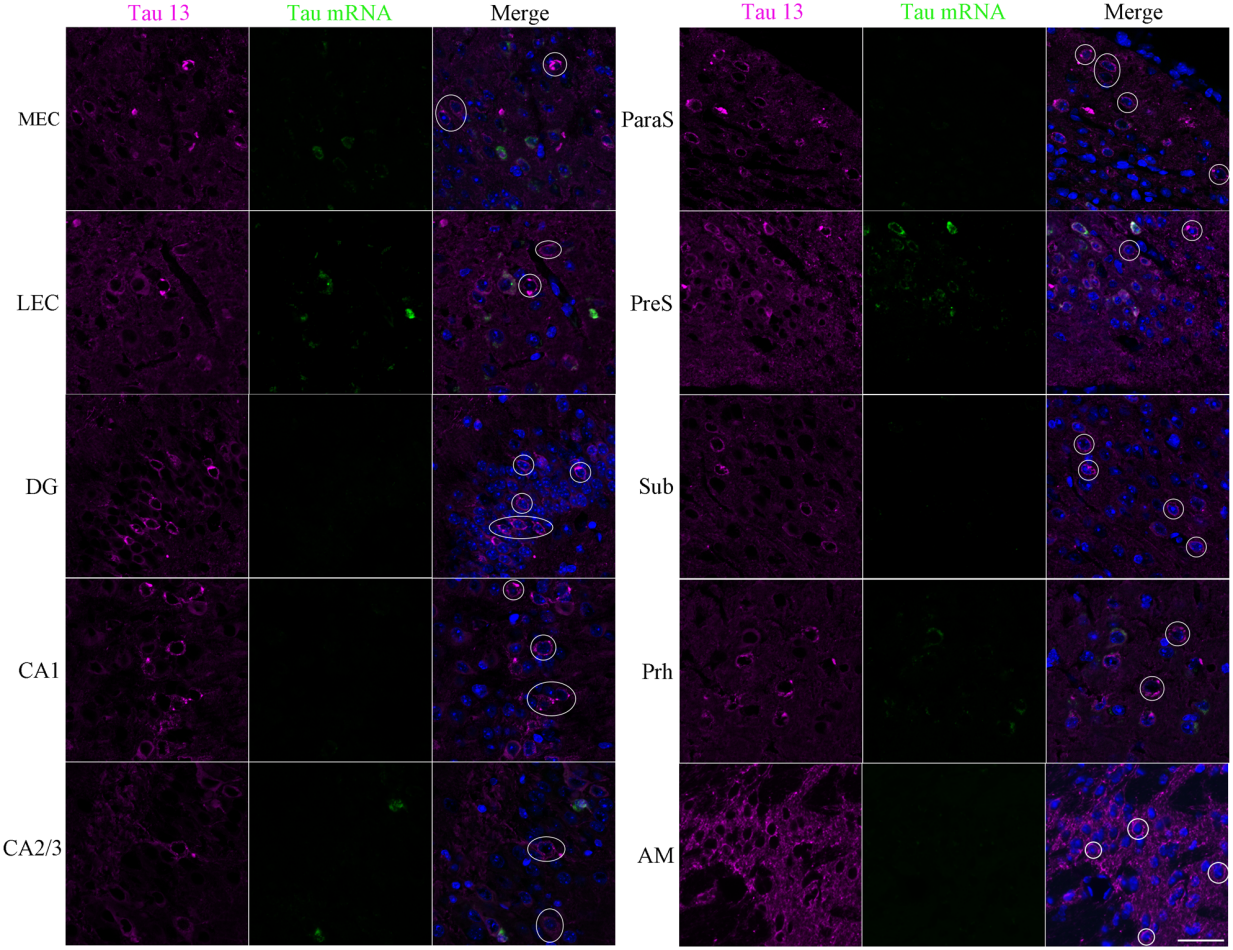
Non cell-autonomous mechanisms may contribute to tau pathology progression outside of the hippocampal formation. Frozen mouse brain sections (10 μm) were subjected to Immuno-FISH as described in the Materials and Methods. Human tau mRNA was labeled with a specific RNA probe (green), and human tau protein was visualized using Tau13 antibody (magenta). The nuclei were counterstained with Hoechst 33342. Z-stack images were taken using an LSM700 confocal microscope. Some cells were identified (white circles or ovals) in most brain regions along the entorhinal cortex-hippocampus circuit, parahippocampal regions and the cortex that were human tau protein positive but human tau mRNA negative. MEC, medial entorhinal cortex; LEC, lateral entorhinal cortex; DG, dentate gyrus; CA, Cornu ammonis; PaS, parasubiculum; PrS, presubiculum; Sub, subiculum; Prh, perirhinal cortex; AM, amygdala. Scale bar = 40 μm.

To investigate whether tau protein accumulation in neurons of the hippocampal formation and other brain regions was due to human tau expression, we employed immuno-FISH to label human tau protein (immunoreactive with anti-human tau antibody Tau13) and human tau mRNA in the same neurons. The results confirmed previous reports that the great majority of human tau immunoreactive cells in the GCL of the EC-Tau mouse were human tau mRNA negative [8], and demonstrated that at least some of human tau positive neurons throughout the EC, HP, Prh, and AM (Fig 5), as well as DI, AI, Pir, and AO (S2 Fig) were human tau mRNA negative supporting the idea that pathological tau can spread non cell-autonomously between brain regions.

### Progressive gliosis in old EC-Tau mice

**Fig 6:**
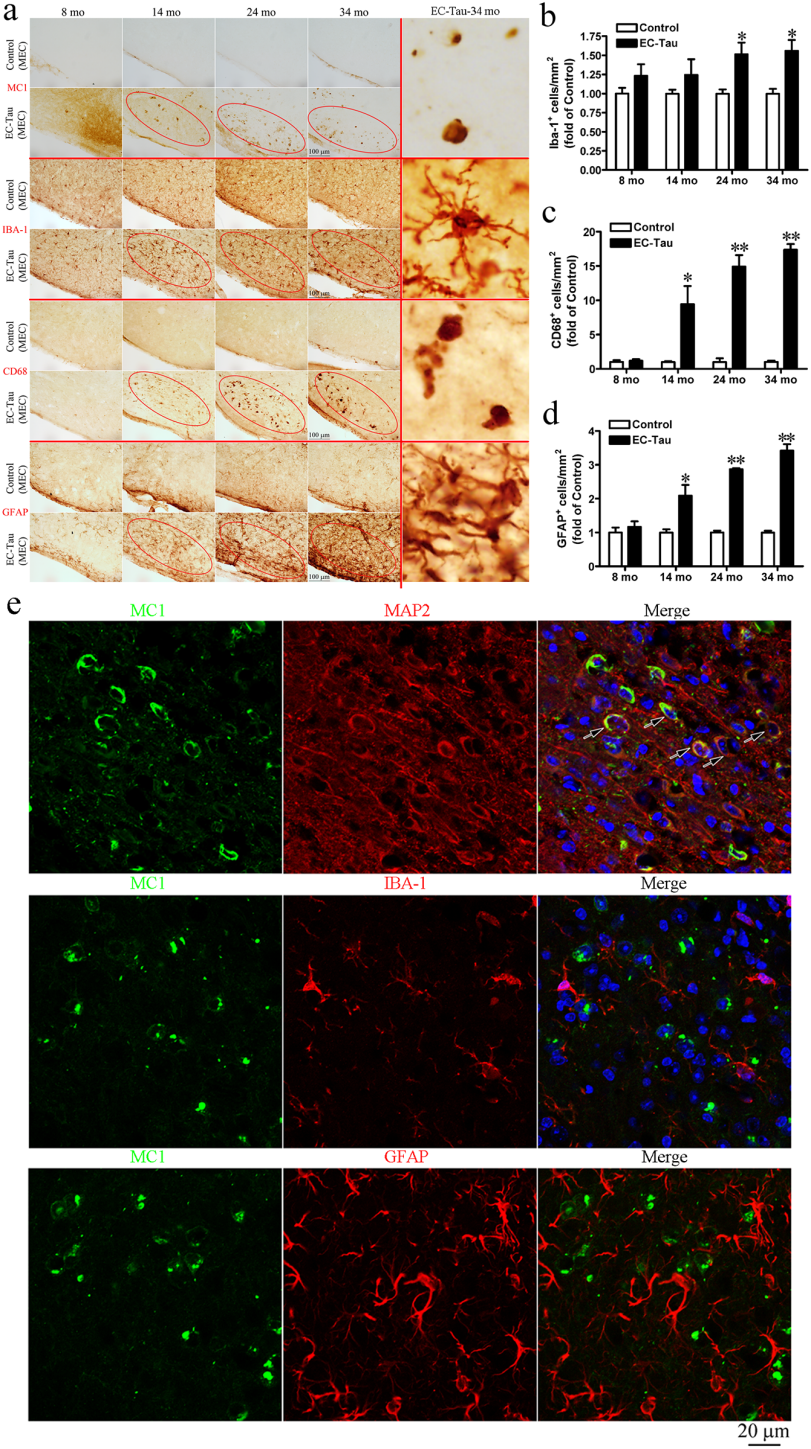
Progressive gliosis is intimately associated with tau pathology in EC-Tau mice. (a) Free-floating sections were incubated with MC1, IBA-1, CD68 or GFAP antibodies. IBA-1^+^ and CD68^+^ microglia and GFAP^+^ astrocytes were found to be recruited to the areas with robust MC1^+^ tau (brown staining inside red ovals). The rightest column of panel a shows the representative high magnification images of MC1, IBA-1, CD68 and GFAP staining in EC-Tau mouse at 34 months of age. MEC, medial entorhinal cortex. Scale bar = 100 μm. (b-d) The quantitation of the number of IBA^+^ cells (b), CD68^+^ cells (c), and GFAP^+^ cells (d) in the MEC of EC-Tau mice (n = 4 animals) and age-matched controls (n = 4 animals). All the data are expressed as mean ± SEM. *P < 0.05, **P < 0.01 *vs*. control (non-parametric Mann-Whitney *U* test). (e) The colocalization of pathological tau (MC1^+^, green) with neurons (MAP2^+^, red), microglia (IBA-1^+^, red) and astrocytes (GFAP^+^, red) was analyzed in 30-mo-old EC-Tau mice using immunofluorescence. The nuclei were counterstained with Hoechst 33342. High magnification Z-stack images showed that the MC1^+^ tau in the MEC was found to be colocalized with MAP2^+^ neurons (arrows), but not with IBA-1^+^ microglia or GFAP^+^ astrocytes. Scale bar = 40 μm.

The activation status of microglia was assessed using antibodies against microglial/macrophage ionized calcium binding adaptor molecule 1 (IBA-1) or CD68; astrocyte activation status was assessed using an antibody against glial fibrillary acidic protein (GFAP) (Fig 6a). Immunohistochemistry showed dramatic changes in microglial morphology from quiescent ramified microglia to activated amoeboid microglia in EC-Tau mice starting at 14 months of age. The distribution of IBA-1^+^ and CD68^+^ microglia was co-incident with the staining pattern of MC1, i.e. it was more prominent in the MEC-II/III (red ovals in Fig 6a). Reactive astrocytes with extended processes and increased synthesis of GFAP protein were also found in the MEC-II/III in EC-Tau mice by 14 months of age (red ovals in Fig 6a). Further, the quantitation of the number of glial markers in the MEC showed that IBA^+^ cells were significantly increased in 24 and 34-mo EC-Tau mice (*P* < 0.05), while both CD68^+^ cells and GFAP^+^ cells were significantly increased in 14, 24 and 34-mo EC-Tau mice compared to age-matched controls (*P* < 0.01) (Figs 6b–6d). Interestingly, activated microglia and reactive astrocytes were found to be associated with tau pathology in the AM (S3 Fig). However, there was no obvious response of microglia or astrocytes in other brain regions compared to age-matched nontransgenic controls (data not shown).

To investigate whether activated microglia and reactive astrocytes accumulate human tau protein, tissues were labeled with fluorescently tagged MC1 antibody, MAP2 antibody (to identify neurons), IBA-1 (to identify microglia), or GFAP (to identify astrocytes). We found that human tau protein was colocalized with MAP2, but not with IBA-1 or GFAP in the MEC of 30-mo-old EC-Tau mice using the z-stack of laser scanning confocal microscopy (Fig 6e), suggesting that human tau accumulates in neurons, rather than microglia or astrocytes.

### Neuronal loss and recognition memory deficits in old EC-Tau mice

**Fig 7:**
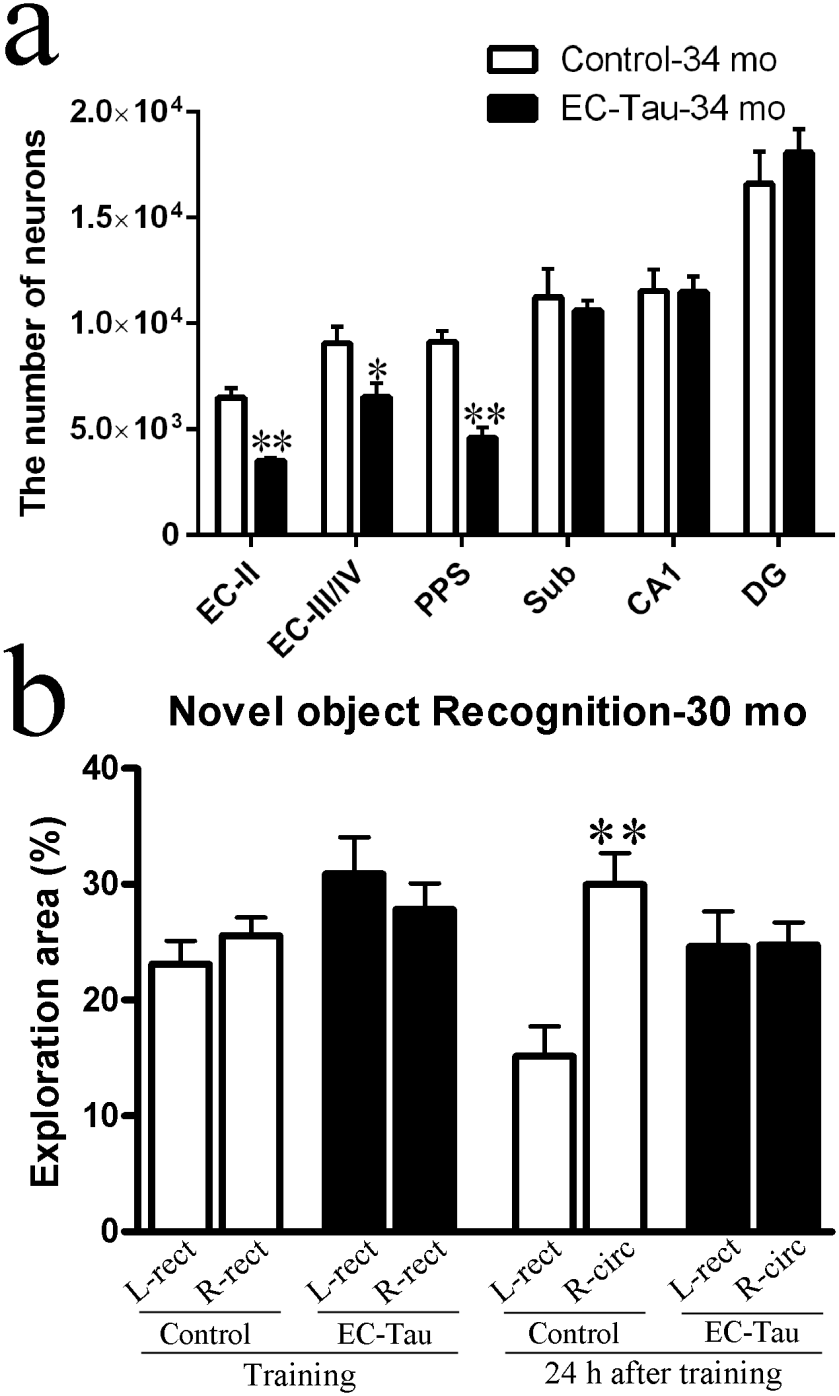
Neuronal loss and recognition memory deficits are observed in old EC-Tau mice. (a) Neuronal loss in old EC-Tau mice. Neurons were stained with NeuN by immunohistochemistry and the number of neurons in different areas of the brain was estimated by a semiquantitative analysis as described in the materials and methods. Significant neuronal loss was detected at 34 months of age in EC-II, EC-III/IV and PPS (n = 5 animals) compared to age matched controls (n = 5 animals). PPS, parasubiculum/presubiculum; Sub, subiculum; CA1, Cornu ammonis 1; DG, dentate gyrus. *p < 0.05, **p < 0.01 *vs*. control (Student’s *t*-test). (b) EC-Tau mice (n = 8 at 30 mo) and littermate controls (n = 8 at 30 mo) were tested in novel object recognition (NOR) as described in the materials and methods. **p < 0.01 *vs*. control (non-parametric Mann-Whitney *U* test).

Previously we only detected significant neuronal loss in the EC-II and PaS in EC-Tau mice at 24, but not at 21 months of age [8]. In this study, we investigated whether neuronal loss worsened with age. By 34 months of age, the number of NeuN^+^ neurons in EC-Tau mice was significantly reduced in the EC-II and PaS/PrS (PPS) compared to age- and gender-matched control mice, and it was also significantly reduced in the EC-III/IV (Fig 7a) (*P* < 0.01), indicating progressive neuronal loss as pathology worsens and spreads through the brain.

NFT load and neuronal loss correlate well with cognitive decline in patients with AD [27–30]. Negligible cognitive deficits were found in EC-Tau mice by 16 months of age in previous reports [9, 20]. By 30 months of age, EC-Tau mice were found to spend much less time with the novel object 24 h after the training, i.e. there are a significant difference in the percent of exploration area in NOR in 30-month-old EC-Tau mice compared to age and gender-matched nontransgenic controls (*p* < 0.05) (Fig 7b), suggesting that long-term object recognition memory was impaired in old EC-Tau mice.

## Discussion

3D volume imaging is an ideal application to explore the spatial distribution of tau pathology in the brain and how it changes with time. Using the iDISCO+ technique in a mouse model with progressive tau pathology, we demonstrate that overt pathological changes generated in the EC and PaS affected new regions of the hippocampal formation and AM by 24 months of age, and the parahippocampal regions including the Prh and other associated neocortical regions as well as the AM by 34 months of age. These data were confirmed using different anti-tau antibodies, and traditional 2D immunolabeling. Using 3D volume imaging we found several areas affected by tau pathology (DI, AI, Pir, AO and AM) in older EC-Tau mice that had not been previously identified by 2D immunostaining. Tau pathology and atrophy has been shown in the AM of human AD patients [6, 31, 32] and the Pir, AO and AM have been proposed to play very important roles in olfaction, emotion and memory in humans. This pathology could explain the olfactory deficits and psychiatric symptoms seen in patients with early AD [6, 31–34].

The tet-O rTg4510 responder line has been reported to be “leaky” in the absence of tTA [9, 35] and the neuropsin-tTA activator line has been shown to drive expression of responder transgenes outside of the hippocampal formation [36, 37]. Although our unactivated tet-O rTg4510 mice had negligible somatodendritic tau pathology detected by IHC, even at the oldest timepoint, some cells did express low levels of human tau as determined by FISH. However, in 30-mo-old EC-Tau mice, we observed cells that were human tau protein+/mRNA- in the Prh, associated neocortical regions (DI, AI, Pir and AO), and in the AM suggesting that some cell to cell spread of tau pathology had occurred in this region. The widespread distribution and abundance of tau pathology in the oldest mice suggests that tau pathology was ramped up as it moved beyond the hippocampal formation, most likely by the templating of pathological tau to human (or mouse) tau expressed at low levels in extrahippocampal neurons. Given that tau expression in humans is widespread, pathology originating in the transentorhinal cortex would be expected to spread through the AD brain in a similar way.

Between 8–14 months of age, we found that activated microglia and reactive astrocytes were enriched in regions of the EC that had developed overt tau pathology. This is consistent with previous findings that glial activation and neuroinflammation were correlated with tau pathology in tau transgenic rodent models and in AD patients [38–40]. A link between tau pathology and the activation of microglia and astrocytes has been reported in mice overexpressing human mutant tau P301S [41, 42], tau R406W [43], tau P301L [8, 44] and disease-modified, truncated tau protein [45, 46]. Although tau inclusions have been found in microglia, astrocytes and oligodendrocytes *in vitro* and/or *in vivo* [8, 40, 47–52], we did not find any MC1^+^ tau inclusions in microglia in the EC-tau line at any age tested. This inconsistency between our data and data from other groups might be due to different detecting methods used by us and others. For example, we blocked all sections with rat anti-mouse FcR block to prevent nonspecific binding of Fc antibody fragments to Fc receptors that are expressed on the surfaces of microglial cells. Other groups did not indicate the use of FcR block in their papers. Also, we used the laser scanning confocal microscope to very carefully look at the colocalization of tau with glial markers in individual slices from z-stack images, whereas other groups did not, or presented the images as a maximum projection of all the slices from the z-stack images. In addition, we used 0.3% Sudan black to quench autofluorescence in the brain sections which can easily lead to misinterpretation of fluorescent images, whereas other groups did not indicate the use of quenching methods. We did observe a few astrocytes that appeared to contain human MC1^+^ tau, similar to what has been reported previously for this line [8]. In general, almost all of the tau inclusions were found in neurons. We conclude that the uptake of pathological tau protein by microglia and/or astrocytes is unlikely to be the major mechanism underlying glia-induced neuroinflammation and the spread of pathological tau in the brain, at least in the EC-tau mouse model. Interestingly, we found that microglia and astrocytes already showed signs of activation by 14 months of age in the EC-tau line, which precedes the time point when tau pathology has matured into thioflavin S positive tangles, and neuronal loss is apparent (data not shown). This is consistent with previously published data that showed that prominent microglial activation appears prior to tangle formation [42]. Microglial activation has recently been suggested to drive tau pathology and contribute to the spread of pathological human wild-type tau in the brain [53] suggesting that neuroinflammation is intimately linked with early cellular events resulting from, or predisposing to tau pathology.

In human AD, NFTs are associated with neuronal loss and cognitive dysfunction [4, 54]. We have previously observed significant neuronal loss at 24 months of age in the EC-II and PaS of EC-Tau mice compared to control mice [8]. In this study, the number of neurons in EC-II and PaS was further reduced in 34-month-old EC-Tau mice, compared to control mice. Additional, significant neuronal loss was also detected in the EC-III/IV and PrS in 34-month-old EC-Tau mice, indicating that neurons may die first at the brain areas with predominant expression of human tau transgene (i.e. EC-II and PaS), followed by the neighboring regions of EC-III/IV and PrS following the spread of pathological tau protein. We did not detect significant neuronal loss in hippocampal or cortical regions even at 34 months which might reflect the paucity of overtly fibillar (thioflavin S positive) tau accumulated in hippocampal neurons. Despite the lack of overt cell loss, synapse loss and altered neuronal activity has been reported in the hippocampus even at considerably earlier ages [8, 9, 20].

Memory dysfunction, especially disruptions of the episodic memory system are among the earliest signs and symptoms of AD, and the most disturbing for patients with AD [54–56]. Episodic memory consists of spatial memory and nonspatial recognition memory. Spatial memory mainly relies on the dominant role of the hippocampus, while recognition memory is mainly contributed by regions such as the MEC, LEC and Prh [57–61]. Using the NOR test, we observed that compared to control mice, the EC-Tau mice explored the novel object for less time 24 h after the training, suggesting that long-term object recognition memory was impaired in old EC-Tau mice. The impairment in object recognition memory is consistent with the abundant tau pathology seen in the LEC and Prh, and the significant neuronal loss in the EC of the oldest EC-Tau mice. Previous studies on tau mouse models with widespread expression of human mutant or wild-type tau in the brain have shown significant cognitive deficits [35, 42, 62–65], however, EC-Tau mice at ages up to 16 months were not cognitively impaired relative to their littermate controls [9, 20]. The authors point out that it is possible that the levels of functionally relevant abnormal tau assemblies were simply not high enough in EC-Tau mice to cause significant behavioral impairments [9]. Extensive aggregation of tau in EC, hippocampus, and especially extrahippocampal regions may be required to cause cognitive decline [29].

## Conclusions

Taken together, we have demonstrated the power of 3D volume imaging using iDISCO+ to observe pathology in deep structures of the mouse brain which has greatly facilitated the tracking of pathology progression through the brain’s anatomical networks. Our findings demonstrate the temporal and spatial relationship between areas as they become affected by pathology, including, for the first time in this mouse model, the neocortical areas. As in human AD, the first signs of cognitive impairment correlate with overt pathology and cell loss in the EC, which in turn correlates with the first appearance of pathology in the neocortex. The EC mouse line is thus a useful model to study the temporal relationship between pathology spread, degeneration and cognition which can help define timepoints when therapeutic interventions may be effective.

## Supporting Information

S1 Fig Tau pathology in neocortical and olfactory areas of old EC-Tau mice. Free-floating horizontal sections from 30-mo-old EC-Tau mice (n = 3 animals) and 32-mo-old Tg4510 control mice (n = 2 animals) were stained with MC1 antibody as described in Materials and Methods. 1, dysgranular insular cortex (DI); 2, agranular insular cortex (AI); 3, piriform cortex (Pir); 4, anterior olfactory area (AO); 5, granular cell layer of olfactory bulb. Scale bar = 500 μm.
(TIF)

S2 Fig Tau pathology spreads to neocortex and olfactory regions in old EC-Tau mice. Frozen sections (10 μm) were subjected to immuno-FISH as described in the Materials and Methods. Human tau protein positive (Tau13^+^) but human tau mRNA negative neurons were found in neocortical regions in 30-mo-old EC-Tau mice, suggesting that tau pathology has the capacity to spread non cell-autonomously in the neocortex. DI, dysgranular insular cortex; AI, agranular insular cortex; Pir, piriform cortex; AO, anterior olfactory bulb. Scale bar = 40 μm.
(TIF)

S3 Fig Gliosis was observed in the amygdala in old EC-Tau mice. Free-floating sections were incubated with IBA-1, CD68 or GFAP antibody. IBA-1^+^ and CD68^+^ microglia and GFAP^+^ astrocytes were found to be recruited to the amygdala in mice with overt tau pathology, but not control mice. Scale bar = 100 μm.
(TIF)

S1 Movie The movie of iDISCO+ immunolabeling of Alexa Fluor 647-CP27 in an 8-mo-old EC-Tau mouse. The movies were generated from 3D rendering as described in Materials and Methods. Different brain regions with tau pathology are indicated by superimposed artificial colors. The regions with no or very little tau immunoreactivity are not colored.
(MP4)

S2 Movie The movie of iDISCO+ immunolabeling of Alexa Fluor 647-CP27 in a 14-mo-old EC-Tau mouse. Different brain regions with tau pathology are indicated by superimposed artificial colors. The regions with no or very little tau immunoreactivity are not colored.
(MP4)

S3 Movie The movie of iDISCO+ immunolabeling of Alexa Fluor 647-CP27 in a 25-mo-old EC-Tau mouse. Different brain regions with tau pathology are indicated by superimposed artificial colors. The regions with no or very little tau immunoreactivity are not colored.
(MP4)

S4 Movie The movie of iDISCO+ immunolabeling of Alexa Fluor 647-CP27 in a 34-mo-old EC-Tau mouse. Different brain regions with tau pathology are indicated by superimposed artificial colors. The regions with no or very little tau immunoreactivity are not colored.
(MP4)

S5 Movie The movie of iDISCO+ immunolabeling of Alexa Fluor 647-CP27 in a 16-mo-old uninduced Tau control mouse (Tg4510, no tTA). There was no positive tau immunoreactivity detected.
(MP4)

S6 Movie The movie of iDISCO+ immunolabeling of Alexa Fluor 647-CP27 in a 33-mo-old Neuropsin tTA (no 4510) control mouse. There was no positive tau immunoreactivity detected.
(MP4)

## References

[1] Ando K, Laborde Q, Lazar A, Godefroy D, Youssef I, Amar M, et al Inside Alzheimer brain with CLARITY: senile plaques, neurofibrillary tangles and axons in 3-D. Acta Neuropathol. 2014;128(3):457–9. 10.1007/s00401-014-1322-y 25069432 PMC4131133

[2] Hama H, Hioki H, Namiki K, Hoshida T, Kurokawa H, Ishidate F, et al ScaleS: an optical clearing palette for biological imaging. Nat Neurosci. 2015;18(10):1518–29. 10.1038/nn.4107 . 26368944

[3] Huang Y, Skwarek-Maruszewska A, Horre K, Vandewyer E, Wolfs L, Snellinx A, et al Loss of GPR3 reduces the amyloid plaque burden and improves memory in Alzheimer's disease mouse models. Sci Transl Med. 2015;7(309):309ra164 10.1126/scitranslmed.aab3492 . 26468326

[4] Braak H, Braak E. Neuropathological stageing of Alzheimer-related changes. Acta Neuropathol. 1991;82(4):239–59. . 1759558 10.1007/BF00308809

[5] Hyman BT, Van Hoesen GW, Damasio AR, Barnes CL. Alzheimer's disease: cell-specific pathology isolates the hippocampal formation. Science. 1984;225(4667):1168–70. . 6474172 10.1126/science.6474172

[6] Braak H, Del Tredici K. The preclinical phase of the pathological process underlying sporadic Alzheimer's disease. Brain. 2015;138(Pt 10):2814–33. 10.1093/brain/awv236 . 26283673

[7] Liu L, Drouet V, Wu JW, Witter MP, Small SA, Clelland C, et al Trans-synaptic spread of tau pathology in vivo. PLoS One. 2012;7(2):e31302 10.1371/journal.pone.0031302 22312444 PMC3270029

[8] de Calignon A, Polydoro M, Suarez-Calvet M, William C, Adamowicz DH, Kopeikina KJ, et al Propagation of tau pathology in a model of early Alzheimer's disease. Neuron. 2012;73(4):685–97. 10.1016/j.neuron.2011.11.033 22365544 PMC3292759

[9] Harris JA, Koyama A, Maeda S, Ho K, Devidze N, Dubal DB, et al Human P301L-mutant tau expression in mouse entorhinal-hippocampal network causes tau aggregation and presynaptic pathology but no cognitive deficits. PLoS One. 2012;7(9):e45881 10.1371/journal.pone.0045881 23029293 PMC3454317

[10] Becker K, Jahrling N, Saghafi S, Weiler R, Dodt HU. Chemical clearing and dehydration of GFP expressing mouse brains. PLoS One. 2012;7(3):e33916 10.1371/journal.pone.0033916 22479475 PMC3316521

[11] Chung K, Deisseroth K. CLARITY for mapping the nervous system. Nat Methods. 2013;10(6):508–13. 10.1038/nmeth.2481 . 23722210

[12] Chung K, Wallace J, Kim SY, Kalyanasundaram S, Andalman AS, Davidson TJ, et al Structural and molecular interrogation of intact biological systems. Nature. 2013;497(7449):332–7. 10.1038/nature12107 23575631 PMC4092167

[13] Erturk A, Becker K, Jahrling N, Mauch CP, Hojer CD, Egen JG, et al Three-dimensional imaging of solvent-cleared organs using 3DISCO. Nat Protoc. 2012;7(11):1983–95. 10.1038/nprot.2012.119 . 23060243

[14] Hama H, Kurokawa H, Kawano H, Ando R, Shimogori T, Noda H, et al Scale: a chemical approach for fluorescence imaging and reconstruction of transparent mouse brain. Nat Neurosci. 2011;14(11):1481–8. 10.1038/nn.2928 . 21878933

[15] Ke MT, Fujimoto S, Imai T. SeeDB: a simple and morphology-preserving optical clearing agent for neuronal circuit reconstruction. Nat Neurosci. 2013;16(8):1154–61. 10.1038/nn.3447 . 23792946

[16] Kuwajima T, Sitko AA, Bhansali P, Jurgens C, Guido W, Mason C. ClearT: a detergent- and solvent-free clearing method for neuronal and non-neuronal tissue. Development. 2013;140(6):1364–8. 10.1242/dev.091844 23444362 PMC3912244

[17] Susaki EA, Tainaka K, Perrin D, Kishino F, Tawara T, Watanabe TM, et al Whole-brain imaging with single-cell resolution using chemical cocktails and computational analysis. Cell. 2014;157(3):726–39. 10.1016/j.cell.2014.03.042 . 24746791

[18] Renier N, Wu Z, Simon DJ, Yang J, Ariel P, Tessier-Lavigne M. iDISCO: a simple, rapid method to immunolabel large tissue samples for volume imaging. Cell. 2014;159(4):896–910. 10.1016/j.cell.2014.10.010 . 25417164

[19] Renier N, Adams EL, Kirst C, Wu Z, Azevedo R, Kohl J, et al Mapping of Brain Activity by Automated Volume Analysis of Immediate Early Genes. Cell. 2016 10.1016/j.cell.2016.05.007 . 27238021 PMC4912438

[20] Polydoro M, Dzhala VI, Pooler AM, Nicholls SB, McKinney AP, Sanchez L, et al Soluble pathological tau in the entorhinal cortex leads to presynaptic deficits in an early Alzheimer's disease model. Acta neuropathologica. 2014;127(2):257–70. 10.1007/s00401-013-1215-5 24271788 PMC3946978

[21] Khan UA, Liu L, Provenzano FA, Berman DE, Profaci CP, Sloan R, et al Molecular drivers and cortical spread of lateral entorhinal cortex dysfunction in preclinical Alzheimer's disease. Nat Neurosci. 2014;17(2):304–11. 10.1038/nn.3606 24362760 PMC4044925

[22] Jicha GA, Berenfeld B, Davies P. Sequence requirements for formation of conformational variants of tau similar to those found in Alzheimer's disease. J Neurosci Res. 1999;55(6):713–23. . 10220112 10.1002/(SICI)1097-4547(19990315)55:6<713::AID-JNR6>3.0.CO;2-G

[23] Schaeren-Wiemers N, Gerfin-Moser A. A single protocol to detect transcripts of various types and expression levels in neural tissue and cultured cells: in situ hybridization using digoxigenin-labelled cRNA probes. Histochemistry. 1993;100(6):431–40. . 7512949 10.1007/BF00267823

[24] Price SR, De Marco Garcia NV, Ranscht B, Jessell TM. Regulation of motor neuron pool sorting by differential expression of type II cadherins. Cell. 2002;109(2):205–16. . 12007407 10.1016/s0092-8674(02)00695-5

[25] Oliveira VC, Carrara RC, Simoes DL, Saggioro FP, Carlotti CG Jr, Covas DT, et al Sudan Black B treatment reduces autofluorescence and improves resolution of in situ hybridization specific fluorescent signals of brain sections. Histology and histopathology. 2010;25(8):1017–24. . 20552552 10.14670/HH-25.1017

[26] Oliveira AM, Hawk JD, Abel T, Havekes R. Post-training reversible inactivation of the hippocampus enhances novel object recognition memory. Learning & memory. 2010;17(3):155–60. 10.1101/lm.1625310 20189960 PMC2832924

[27] Gomez-Isla T, Price JL, McKeel DW Jr, Morris JC, Growdon JH, Hyman BT. Profound loss of layer II entorhinal cortex neurons occurs in very mild Alzheimer's disease. The Journal of neuroscience: the official journal of the Society for Neuroscience. 1996;16(14):4491–500. . 8699259 10.1523/JNEUROSCI.16-14-04491.1996 PMC6578866

[28] Gomez-Isla T, Hollister R, West H, Mui S, Growdon JH, Petersen RC, et al Neuronal loss correlates with but exceeds neurofibrillary tangles in Alzheimer's disease. Annals of neurology. 1997;41(1):17–24. 10.1002/ana.410410106 . 9005861

[29] Giannakopoulos P, Herrmann FR, Bussiere T, Bouras C, Kovari E, Perl DP, et al Tangle and neuron numbers, but not amyloid load, predict cognitive status in Alzheimer's disease. Neurology. 2003;60(9):1495–500. . 12743238 10.1212/01.wnl.0000063311.58879.01

[30] Serrano-Pozo A, Frosch MP, Masliah E, Hyman BT. Neuropathological alterations in Alzheimer disease. Cold Spring Harbor perspectives in medicine. 2011;1(1):a006189 10.1101/cshperspect.a006189 22229116 PMC3234452

[31] Horinek D, Petrovicky P, Hort J, Krasensky J, Brabec J, Bojar M, et al Amygdalar volume and psychiatric symptoms in Alzheimer's disease: an MRI analysis. Acta Neurol Scand. 2006;113(1):40–5. 10.1111/j.1600-0404.2006.00540.x . 16367898

[32] Poulin SP, Dautoff R, Morris JC, Barrett LF, Dickerson BC, Alzheimer's Disease Neuroimaging I. Amygdala atrophy is prominent in early Alzheimer's disease and relates to symptom severity. Psychiatry Res. 2011;194(1):7–13. 10.1016/j.pscychresns.2011.06.014 21920712 PMC3185127

[33] Doty RL, Reyes PF, Gregor T. Presence of both odor identification and detection deficits in Alzheimer's disease. Brain Res Bull. 1987;18(5):597–600. . 3607528 10.1016/0361-9230(87)90129-8

[34] Devanand DP, Michaels-Marston KS, Liu X, Pelton GH, Padilla M, Marder K, et al Olfactory deficits in patients with mild cognitive impairment predict Alzheimer's disease at follow-up. Am J Psychiatry. 2000;157(9):1399–405. . 10964854 10.1176/appi.ajp.157.9.1399

[35] Santacruz K, Lewis J, Spires T, Paulson J, Kotilinek L, Ingelsson M, et al Tau suppression in a neurodegenerative mouse model improves memory function. Science. 2005;309(5733):476–81. 10.1126/science.1113694 16020737 PMC1574647

[36] Yasuda M, Mayford MR. CaMKII activation in the entorhinal cortex disrupts previously encoded spatial memory. Neuron. 2006;50(2):309–18. 10.1016/j.neuron.2006.03.035 . 16630840

[37] Yetman MJ, Lillehaug S, Bjaalie JG, Leergaard TB, Jankowsky JL. Transgene expression in the Nop-tTA driver line is not inherently restricted to the entorhinal cortex. Brain Struct Funct. 2015 10.1007/s00429-015-1040-9 25869275 PMC4605847

[38] Sheng JG, Mrak RE, Griffin WS. Glial-neuronal interactions in Alzheimer disease: progressive association of IL-1alpha+ microglia and S100βετα+ astrocytes with neurofibrillary tangle stages. J Neuropathol Exp Neurol. 1997;56(3):285–90. . 9056542

[39] Ballatore C, Lee VM, Trojanowski JQ. Tau-mediated neurodegeneration in Alzheimer's disease and related disorders. Nat Rev Neurosci. 2007;8(9):663–72. 10.1038/nrn2194 . 17684513

[40] Zilka N, Kazmerova Z, Jadhav S, Neradil P, Madari A, Obetkova D, et al Who fans the flames of Alzheimer's disease brains? Misfolded tau on the crossroad of neurodegenerative and inflammatory pathways. J Neuroinflammation. 2012;9:47 10.1186/1742-2094-9-47 22397366 PMC3334709

[41] Bellucci A, Westwood AJ, Ingram E, Casamenti F, Goedert M, Spillantini MG. Induction of inflammatory mediators and microglial activation in mice transgenic for mutant human P301S tau protein. Am J Pathol. 2004;165(5):1643–52. 10.1016/S0002-9440(10)63421-9 15509534 PMC1618683

[42] Yoshiyama Y, Higuchi M, Zhang B, Huang SM, Iwata N, Saido TC, et al Synapse loss and microglial activation precede tangles in a P301S tauopathy mouse model. Neuron. 2007;53(3):337–51. 10.1016/j.neuron.2007.01.010 . 17270732

[43] Ikeda M, Shoji M, Kawarai T, Kawarabayashi T, Matsubara E, Murakami T, et al Accumulation of filamentous tau in the cerebral cortex of human tau R406W transgenic mice. Am J Pathol. 2005;166(2):521–31. 10.1016/S0002-9440(10)62274-2 15681835 PMC1602315

[44] Sasaki A, Kawarabayashi T, Murakami T, Matsubara E, Ikeda M, Hagiwara H, et al Microglial activation in brain lesions with tau deposits: comparison of human tauopathies and tau transgenic mice TgTauP301L. Brain Res. 2008;1214:159–68. 10.1016/j.brainres.2008.02.084 . 18457819

[45] Zilka N, Stozicka Z, Kovac A, Pilipcinec E, Bugos O, Novak M. Human misfolded truncated tau protein promotes activation of microglia and leukocyte infiltration in the transgenic rat model of tauopathy. J Neuroimmunol. 2009;209(1–2):16–25. 10.1016/j.jneuroim.2009.01.013 . 19232747

[46] Stozicka Z, Zilka N, Novak P, Kovacech B, Bugos O, Novak M. Genetic background modifies neurodegeneration and neuroinflammation driven by misfolded human tau protein in rat model of tauopathy: implication for immunomodulatory approach to Alzheimer's disease. J Neuroinflammation. 2010;7:64 10.1186/1742-2094-7-64 20937161 PMC2958906

[47] Majerova P, Zilkova M, Kazmerova Z, Kovac A, Paholikova K, Kovacech B, et al Microglia display modest phagocytic capacity for extracellular tau oligomers. J Neuroinflammation. 2014;11:161 10.1186/s12974-014-0161-z 25217135 PMC4172893

[48] Luo W, Liu W, Hu X, Hanna M, Caravaca A, Paul SM. Microglial internalization and degradation of pathological tau is enhanced by an anti-tau monoclonal antibody. Sci Rep. 2015;5:11161 10.1038/srep11161 26057852 PMC4460904

[49] Chin SS, Goldman JE. Glial inclusions in CNS degenerative diseases. J Neuropathol Exp Neurol. 1996;55(5):499–508. . 8627339 10.1097/00005072-199605000-00001

[50] Nakano I, Iwatsubo T, Otsuka N, Kamei M, Matsumura K, Mannen T. Paired helical filaments in astrocytes: electron microscopy and immunohistochemistry in a case of atypical Alzheimer's disease. Acta Neuropathol. 1992;83(3):228–32. . 1557954 10.1007/BF00296783

[51] Nishimura M, Tomimoto H, Suenaga T, Namba Y, Ikeda K, Akiguchi I, et al Immunocytochemical characterization of glial fibrillary tangles in Alzheimer's disease brain. Am J Pathol. 1995;146(5):1052–8. 7747799 PMC1869277

[52] Asai H, Ikezu S, Tsunoda S, Medalla M, Luebke J, Haydar T, et al Depletion of microglia and inhibition of exosome synthesis halt tau propagation. Nat Neurosci. 2015;18(11):1584–93. 10.1038/nn.4132 . 26436904 PMC4694577

[53] Maphis N, Xu G, Kokiko-Cochran ON, Jiang S, Cardona A, Ransohoff RM, et al Reactive microglia drive tau pathology and contribute to the spreading of pathological tau in the brain. Brain. 2015;138(Pt 6):1738–55. 10.1093/brain/awv081 25833819 PMC4542622

[54] Gold CA, Budson AE. Memory loss in Alzheimer's disease: implications for development of therapeutics. Expert review of neurotherapeutics. 2008;8(12):1879–91. 10.1586/14737175.8.12.1879 19086882 PMC2655107

[55] Caterini F, Della Sala S, Spinnler H, Stangalino C, Tumbull OH. Object recognition and object orientation in Alzheimer's disease. Neuropsychology. 2002;16(2):146–55. . 11949706

[56] Budson AE, Price BH. Memory dysfunction. The New England journal of medicine. 2005;352(7):692–9. 10.1056/NEJMra041071 . 15716563

[57] Buckley MJ. The role of the perirhinal cortex and hippocampus in learning, memory, and perception. The Quarterly journal of experimental psychology B, Comparative and physiological psychology. 2005;58(3–4):246–68. 10.1080/02724990444000186 . 16194968

[58] Sauvage MM, Beer Z, Ekovich M, Ho L, Eichenbaum H. The caudal medial entorhinal cortex: a selective role in recollection-based recognition memory. The Journal of neuroscience: the official journal of the Society for Neuroscience. 2010;30(46):15695–9. 10.1523/JNEUROSCI.4301-10.2010 21084625 PMC3073554

[59] Wilson DI, Langston RF, Schlesiger MI, Wagner M, Watanabe S, Ainge JA. Lateral entorhinal cortex is critical for novel object-context recognition. Hippocampus. 2013;23(5):352–66. 10.1002/hipo.22095 23389958 PMC3648979

[60] Wilson DI, Watanabe S, Milner H, Ainge JA. Lateral entorhinal cortex is necessary for associative but not nonassociative recognition memory. Hippocampus. 2013;23(12):1280–90. 10.1002/hipo.22165 23836525 PMC4030623

[61] Deshmukh SS, Johnson JL, Knierim JJ. Perirhinal cortex represents nonspatial, but not spatial, information in rats foraging in the presence of objects: comparison with lateral entorhinal cortex. Hippocampus. 2012;22(10):2045–58. 10.1002/hipo.22046 22987681 PMC3870144

[62] Tatebayashi Y, Miyasaka T, Chui DH, Akagi T, Mishima K, Iwasaki K, et al Tau filament formation and associative memory deficit in aged mice expressing mutant (R406W) human tau. Proceedings of the National Academy of Sciences of the United States of America. 2002;99(21):13896–901. 10.1073/pnas.202205599 12368474 PMC129794

[63] Taniguchi T, Doe N, Matsuyama S, Kitamura Y, Mori H, Saito N, et al Transgenic mice expressing mutant (N279K) human tau show mutation dependent cognitive deficits without neurofibrillary tangle formation. FEBS letters. 2005;579(25):5704–12. 10.1016/j.febslet.2005.09.047 . 16219306

[64] Kimura T, Yamashita S, Fukuda T, Park JM, Murayama M, Mizoroki T, et al Hyperphosphorylated tau in parahippocampal cortex impairs place learning in aged mice expressing wild-type human tau. The EMBO journal. 2007;26(24):5143–52. 10.1038/sj.emboj.7601917 18007595 PMC2140104

[65] Sydow A, Van der Jeugd A, Zheng F, Ahmed T, Balschun D, Petrova O, et al Tau-induced defects in synaptic plasticity, learning, and memory are reversible in transgenic mice after switching off the toxic Tau mutant. The Journal of neuroscience: the official journal of the Society for Neuroscience. 2011;31(7):2511–25. 10.1523/JNEUROSCI.5245-10.2011 . 21325519 PMC6623704

