# Supplementary figures and images for: 3D Visualization of the Temporal and Spatial Spread of Tau Pathology Reveals Extensive Sites of Tau Accumulation Associated with Neuronal Loss and Recognition Memory Deficit in Aged Tau Transgenic Mice
Source: PLoS One. 2016 Jul 28;11(7):e0159463. doi: 10.1371/journal.pone.0159463 (PMC4965059; doi:10.1371/journal.pone.0159463)

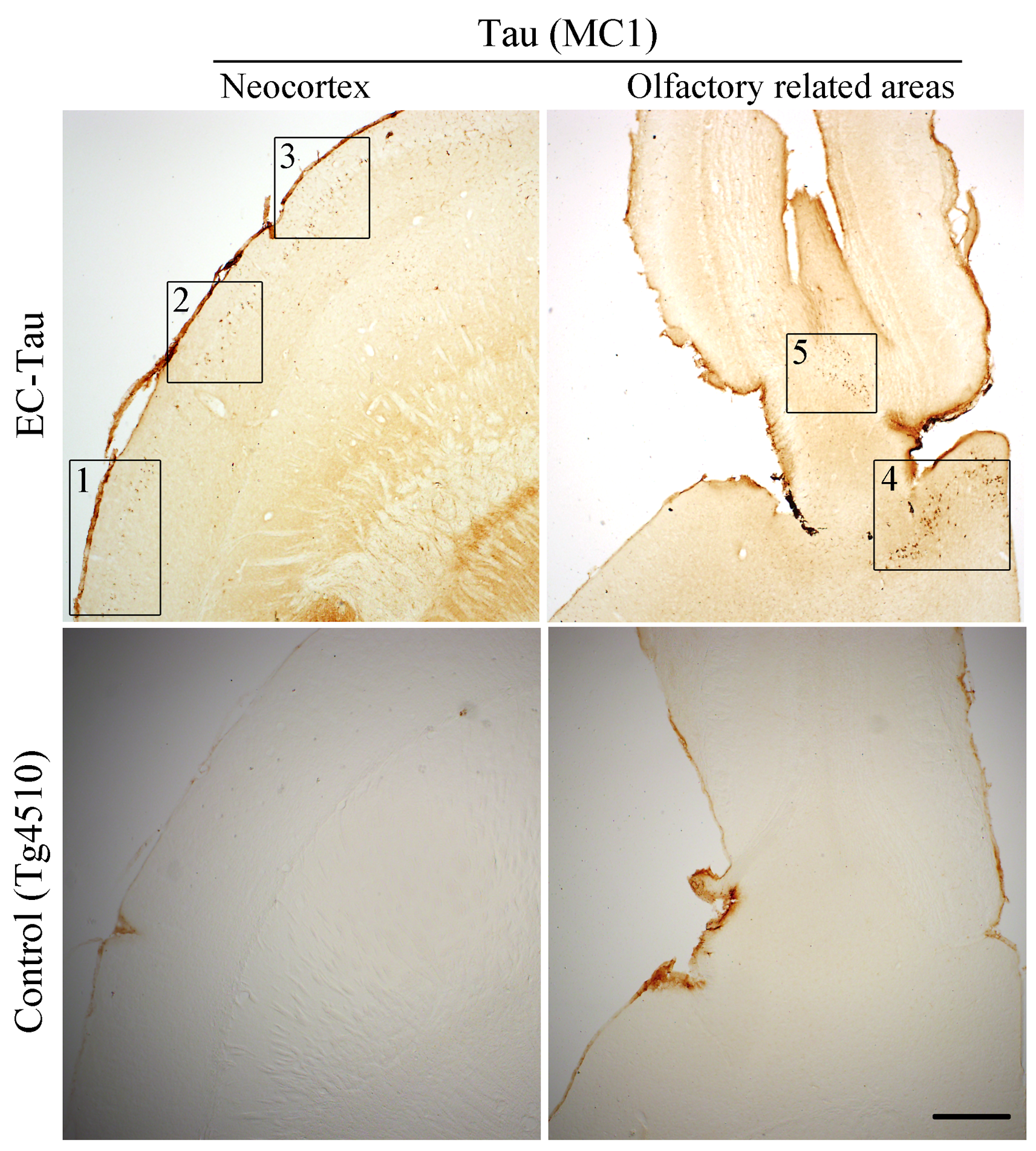

Supplement: S1 Fig — Free-floating horizontal sections from 30-mo-old EC-Tau mice (n = 3 animals) and 32-mo-old Tg4510 control mice (n = 2 animals) were stained with MC1 antibody as described in Materials and Methods. 1, dysgranular insular cortex (DI); 2, agranular insular cortex (AI); 3, piriform cortex (Pir); 4, anterior olfactory area (AO); 5, granular cell layer of olfactory bulb. Scale bar = 500 μm. (TIF) [file pone.0159463.s001.tif]

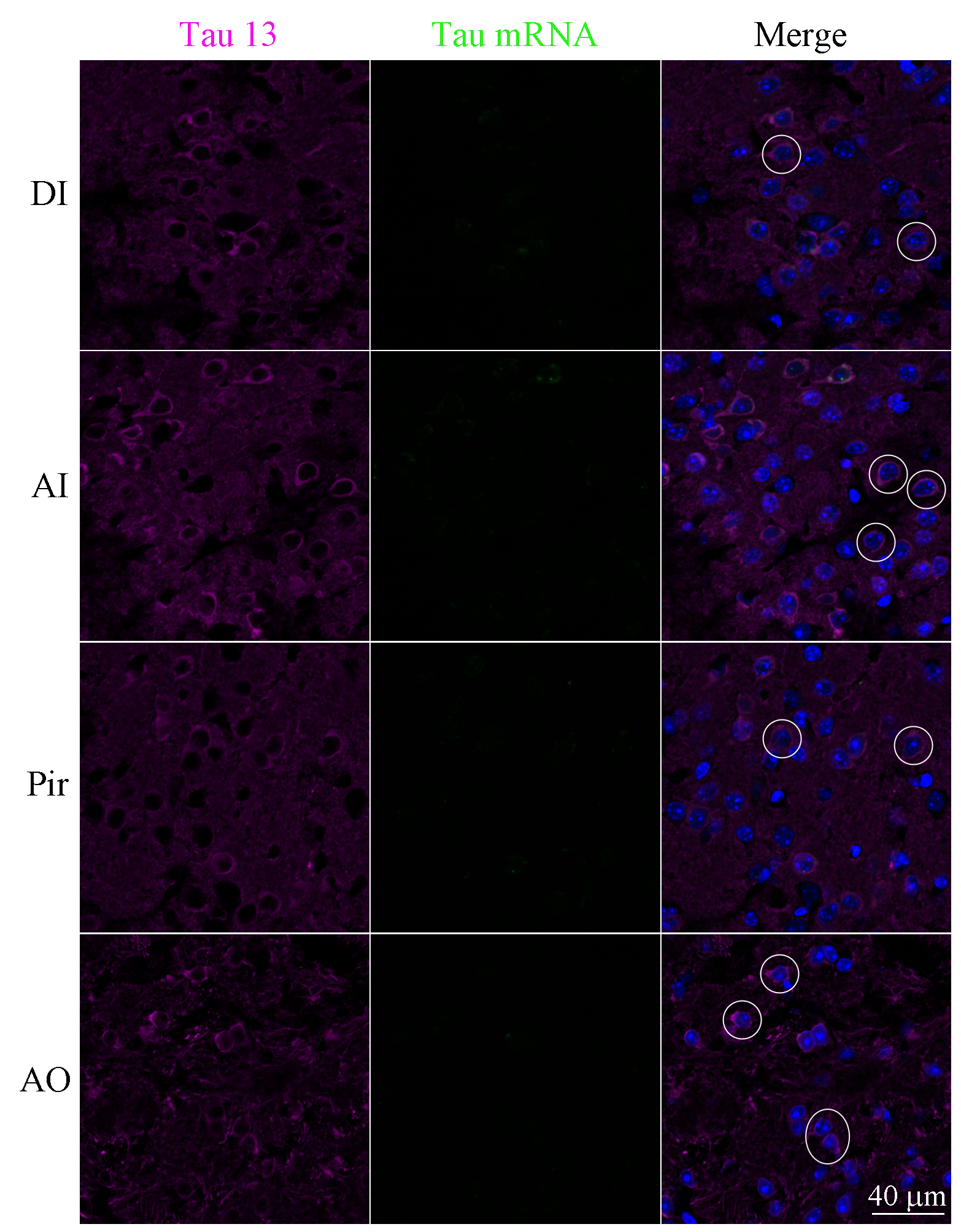

Supplement: S2 Fig — Frozen sections (10 μm) were subjected to immuno-FISH as described in the Materials and Methods. Human tau protein positive (Tau13+) but human tau mRNA negative neurons were found in neocortical regions in 30-mo-old EC-Tau mice, suggesting that tau pathology has the capacity to spread non cell-autonomously in the neocortex. DI, dysgranular insular cortex; AI, agranular insular cortex; Pir, piriform cortex; AO, anterior olfactory bulb. Scale bar = 40 μm. (TIF) [file pone.0159463.s002.tif]

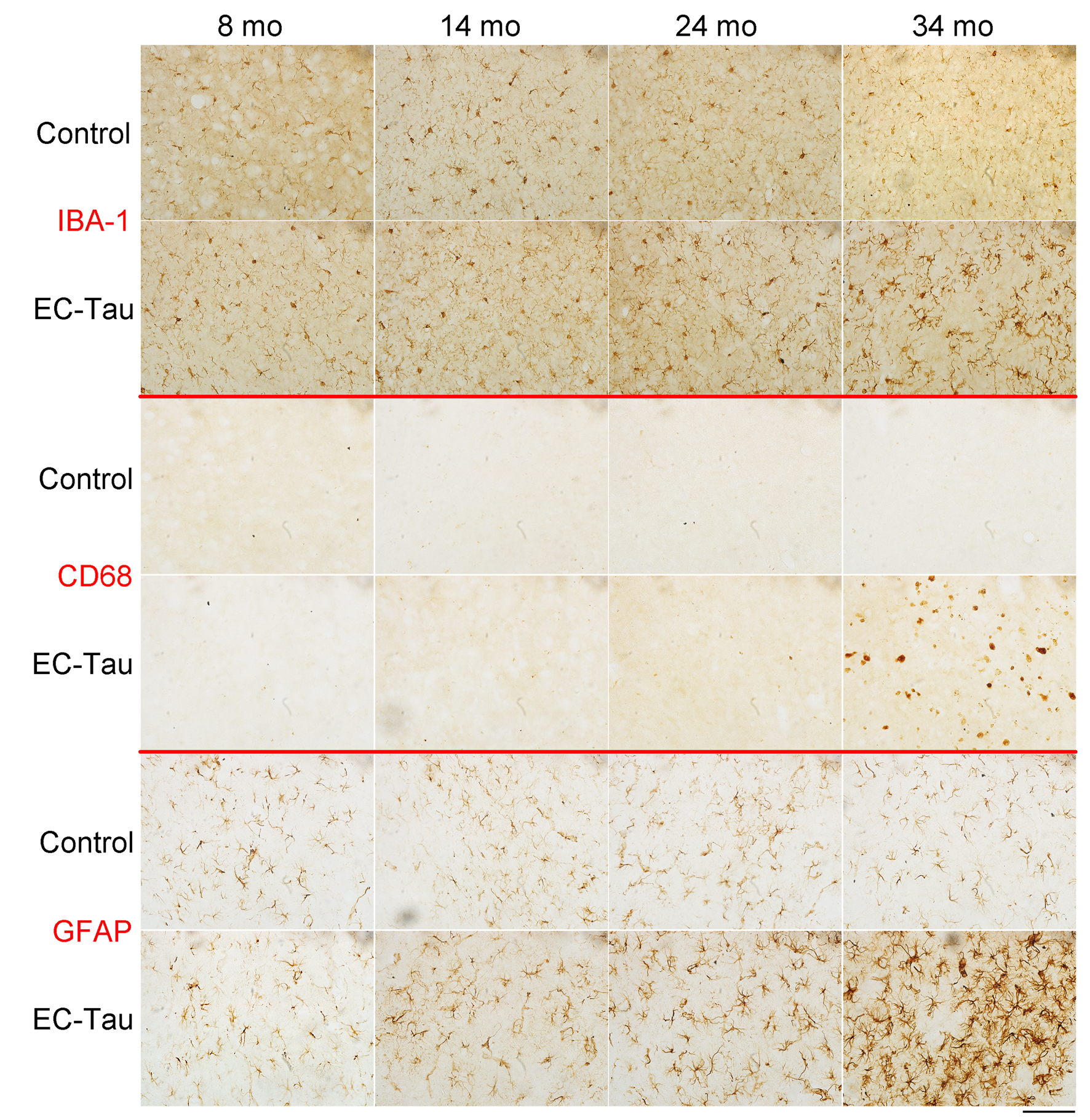

Supplement: S3 Fig — Free-floating sections were incubated with IBA-1, CD68 or GFAP antibody. IBA-1+ and CD68+ microglia and GFAP+ astrocytes were found to be recruited to the amygdala in mice with overt tau pathology, but not control mice. Scale bar = 100 μm. (TIF) [file pone.0159463.s003.tif]
